# Solid-State Construction of CuO–Cu_2_O@C with Synergistic Effects of Pseudocapacity and Carbon Coating for Enhanced Electrochemical Lithium Storage

**DOI:** 10.3390/nano14171378

**Published:** 2024-08-23

**Authors:** Guifen Du, Piyu Gong, Chuansheng Cui, Lei Wang, Changhua An

**Affiliations:** 1 Department of Chemistry, Liaocheng University, Liaocheng 252059, China; duguifen163@163.com (G.D.); 18315756459@163.com (P.G.); cuichuansheng@lcu.edu.cn (C.C.); 2 School of Chemistry and Chemical Engineering, Tianjin University of Technology, Tianjin 300384, China

**Keywords:** salt bath, mixed-valent copper oxides, lithium-ion battery, anode materials, pseudocapacitive behavior

## Abstract

The pseudocapacitive effect can improve the electrochemical lithium storage capacity at high-rate current density. However, the cycle stability is still unsatisfactory. To overcome this issue, a multivalent oxide with a carbon coating represents a plausible technique. In this work, a CuO–Cu_2_O@C composite has been constructed by a one-step bilayer salt-baking process and utilized as anode material for lithium-ion batteries. At a current density of 2.0 A g^−1^, the as-prepared composite delivered a stable discharge capacity of 431.8 mA h g^−1^ even after 600 cycles. The synergistic effects of the multivalence, the pseudocapacitive contribution from copper, and the carbon coating contribute to the enhanced electrochemical lithium storage performance. Specifically, the existence of cuprous suboxide improves the electrochemical conductivity, the pseudocapacitive effect enhances the lithium storage capacity, and the presence of carbon ensures cycle stability. The testing results show that CuO–Cu_2_O@C composite has broad application prospects in portable energy storage devices. The present work provides an instructive precedent for the preparation of transition metal oxides with controllable electronic states and excellent electrochemical performance.

## 1. Introduction

Today, environmental-friendly lithium-ion batteries (LIBs) with high energy density have become increasingly important with the rapid development of advanced consumer electronics [1,2,3]. However, the energy density of traditional graphite-based LIBs is greatly limited, which cannot meet the long-distance operation of electric vehicles [4]. Therefore, the design of suitable anode materials to replace graphite for the next generation of LIBs is important. As an anode material, transition metal oxides (TMOs) has a high theoretical specific capacity in the multi-electron reaction mechanism with Li [5]. M_x_O_y_ has been considered as one type of important anode material. At present, TMOs, including Mn_3_O_4_, V_2_O_5_, Fe_2_O_3_, MoO_3,_ and CuO, that possess high energy density have been successfully prepared [6,7,8,9,10]. Among them, copper (I/II) oxide is particularly attractive in LIBs due to its good electrochemical performance. The theoretical specific capacity of copper oxide reaches 670 mA h g^−1^ [11], and the non-toxic and simple synthesis process makes it a potential negative material for LIBs. However, large volume expansion and low electronic conductivity of CuO will emerge in the electrochemical process, which impedes the application of the CuO electrodes [12,13]. In order to improve the lithium storage performance of copper oxide, adding carbon materials and forming mixed-valence complexes are good alternative strategies [14].

Recently, Song et al. reported a low-temperature heat treatment technique to grow 3D Cu_2_O anode material directly on copper foam; the formed Cu_2_O showed a high specific capacity and exceptional cyclic performance with negligible degradation after 200 cycles [15]. Xu et al. produced ultrafine CuO nanoparticles within porous carbon octahedral (CuO@C); the interconnected channels of the carbon matrix provided rapid electrolyte and electron transfer and lower volume expansion during cycling [11]. Sun et al. synthesized a Cu–MOF precursor and then calcined it at various temperatures to produce electrode materials [16]. Lin et al. described that porous Cu_2_O–CuO–C/Cu was prepared by a solvothermal approach and annealing in air, which could be utilized as the anode of LIBs and presented a discharge capacity of 1321 mA h g^−1^ at 0.1 A g^−1^ after 500 cycles [17].

Although copper oxide/cuprous oxide have made great improvements in electrochemical performance, they still suffer from capacity attenuation due to volume expansion. Carbon coating is effective in enhancing the conductivity and structural stability of anode materials and improving the cycling stability of formed LIBs [18]. Furthermore, the mixed-valence copper oxide (Cu_2_O/CuO) has been used in many fields [19]. Therefore, many synthetic methods for preparing mixed-valence copper oxide have been explored, such as hot plates, microwave ovens, and heated tungsten wire. Currently, there is extensive research on copper-containing transition metal oxides and their composites for use in LIBs, resulting in significant advancements [4]. Although binary metal compounds and carbon complexes have been reported using the solid-state fabrication method [18], it has yet to be employed for the synthesis of CuO, Cu_2_O, and C complexes [19]. The biggest difficulty is to control the temperature so that the synthesized composite can contain copper oxides with different valence states.

In this work, a salt bath baking method was utilized for the preparation of mixed-valence copper oxide. A CuO–Cu_2_O@C composite can be prepared at 325 °C. The internal molten KBr medium provides the decomposition condition of copper acetate, and the externally sealed NaCl layer is used as the closed and protective medium to prevent the consumption of newly formed carbon during the annealing. Electrochemical tests show that the formed CuO–Cu_2_O@C composite endows better electrochemical performance. The result indicates that this bilayer salt bath baking method is conducive to the synthesis of mixed valence oxides@C composite, which presents a new design strategy for mixed-valence oxide-based anode material for the preparation of high-performance LIBs.

## 2. Materials and Methods

### 2.1. Synthesis of Curled Sunflower Shaped CuO–Cu_2_O@C Composite Anode Material

A CuO–Cu_2_O@C composite was prepared using a bilayer salt bath baking method. Briefly, 0.4 g of copper acetate and 1.0 g of potassium bromide were fully ground. The obtained powder was extruded into a small column with a diameter of 1 cm (Column A). Subsequently, the prepared small column was encapsulated with the comminuted NaCl powder and took shape into a large-size cylinder with a diameter of 2.0 cm (Column B). Then, the NaCl-encapsulated reactant was heated to 325 °C at a heating rate of 5 °C min^−1^ in the air, holding for 1 h. Finally, Column B was washed with water thoroughly and dried. The obtained sample is CuO–Cu_2_O@C composite. For comparison, the CuO@C composite was prepared at a heating temperature of 450 °C, while other conditions remained unchanged. Because KBr has a greater solubility than NaCl in water, the obtained product can be easily cleaned. It is worth noting that the bilayer salts of KBr and NaCl will not affect the purity of the product and are easy to remove.

### 2.2. Materials Characterization

The structure of the formed sample was confirmed by a Smartlab X-ray powder diffraction diffractometer (XRD, Rigaku Corporation, Tokyo, Japan) using Cu Kα radiation (λ = 0.15406 nm). The morphology, size, and elemental distribution of the product were determined using GX4 field emission scanning electron microscopy (FE-SEM, FIB-SEM Eindhoven, The Netherlands) equipped with Bruker energy dispersive spectroscopy (EDS, Karlsruhe, Germany) and a Talos F200x high-resolution transmission electron microscopy (HRTEM, FEI Company, Eindhoven, The Netherlands). The structure features of the formed products were analyzed on an Invia Raman microscope with an excitation line of 532 nm (Renishaw, London, UK) and a Nicolet 6700 FT-IR spectrometer (Thermo Fisher Scientific, Waltham, MA, USA). The composition and elemental state of the obtained samples were tested on an EACALAB 250 X-ray photoelectron spectroscopy (XPS, Thermo Fisher Scientific, Waltham, MA, USA). The obtained XPS data were corrected with the C (1s) peak at 284.6 eV as an internal standard. The specific surface area and aperture characteristics of the product were tested using an autosorb IQ-C nitrogen adsorption/desorption instrument. Thermogravimetric analysis (TGA) was carried out in an oxygen atmosphere using the STA449F5-QMS403D synchronous thermal analyzer (Netzsch, Selb, Freistaat Bayern, Germany). The sample was placed in an Al_2_O_3_ crucible and heated from room temperature to 800 °C.

### 2.3. Electrochemical Measurement

CR2032 coin-type half batteries were utilized for the electrochemical measurement of the formed samples. In order to prepare the working electrode, the active material, Super-P carbon and sodium carboxymethyl cellulose adhesive (7:2:1, weight%), was ground and mixed evenly, then the obtained slurry was daubed on the copper foil and dried at 100 °C. The mass loading of active material on the working electrode is about 1.0 mg cm^−2^. The reference electrode is a lithium disk; the separator is Celgard 2400 film; and the electrolyte used in this work is 1 mol L^−1^ LiPF_6_. Battery assembly was conducted in an argon-filled glove box. The performance test of the formed LIBs was executed on the LAND CT2001A system (Wuhan Land Electronics Co., Ltd., Wuhan, China) at room temperature. The activation of the assembled LIBs was implemented at 0.1 A g^−1^ for the first three cycles. The cyclic voltammetry (CV) plots from 0 to 3.0 V and the electrochemical impedance spectroscopy (EIS) plots (test range from 0.01 Hz to 100 kHz) were recorded using the GAMRY Reference 3000+ electrochemical workstation (Philadelphia, PA, USA).

## 3. Results and Discussion

### 3.1. Characterization of the Formed Samples

**Figure 1:**
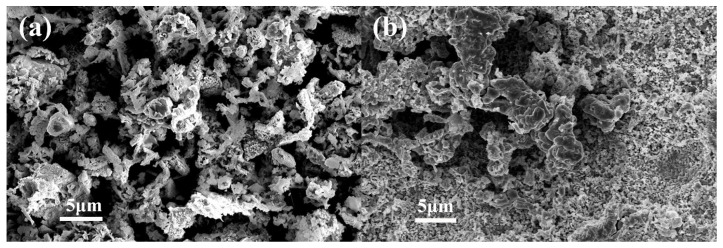
SEM images of CuO–Cu_2_O@C composite (**a**) and CuO@C composite (**b**).

The morphology and size of the product were investigated. FE-SEM images show that the product obtained at 325 °C presents a curled sunflower shape. The curled sunflower shaped microstructures are composed of many different nanopyramids with sizes of 200–500 nm (Figure 1a). As the heating temperature increases to 450 °C, porous microstructures are constructed by irregular particle accumulation (Figure 1b).

**Figure 2:**
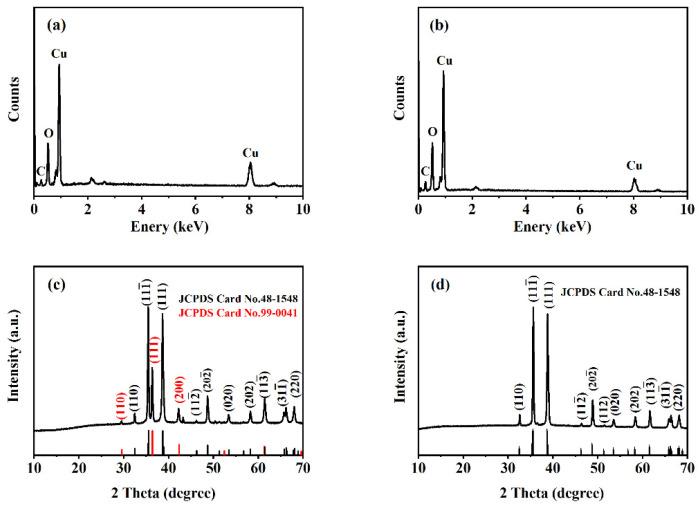
EDS and XRD patterns of CuO–Cu_2_O@C composite (**a**,**c**) and CuO@C composite (**b**,**d**).

The elemental composition and structure of two formed samples were analyzed. EDS reveals that the obtained products are composed of Cu, O, and C (Figure 2a,b). For the sample prepared at a salt bath baking temperature of 325 °C (Figure 2c), the diffraction peak at 32.52, 35.47, 38.92, 48.71, 53.52, 58.27, 61.55, 66.31, 68.17 degrees can be attributed to the (110), (11-1), (111), (20-2), (020), (202), (11-3), (31-1) and (220) planes of monoclinic CuO crystal (PDF # 48-1548), and the diffraction peak at 29.52, 36.31, 42.25 degrees can be ascribed to the (110), (111), and (200) planes of cubic Cu_2_O crystal (PDF # 99-0041). The XRD pattern proves that a CuO–Cu_2_O composite can be prepared. For the sample prepared at a salt bath baking temperature of 450 °C (Figure 2d), the main diffraction peaks correspond to the monoclinic CuO (PDF # 48-1548). XRD results suggest that the curled sunflower-shaped microstructure has high crystallinity and phase purity and proves the formation of a CuO–Cu_2_O composite. 

**Figure 3:**
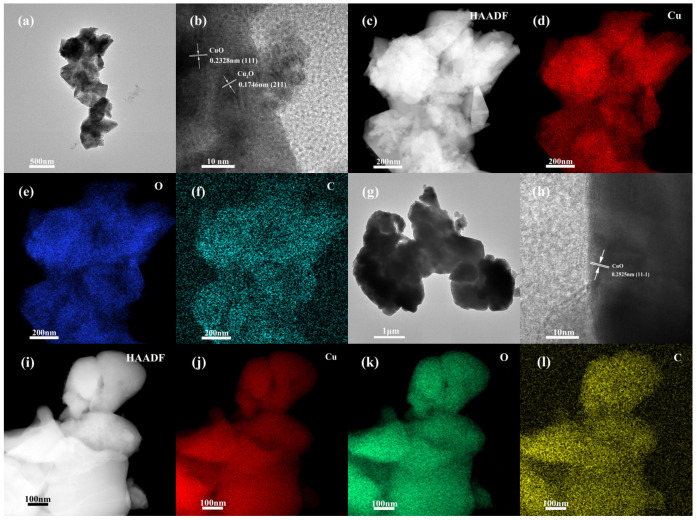
TEM, HRTEM images, and elemental mappings of CuO–Cu_2_O@C composite (**a**–**f**) and CuO@C composite (**g**–**l**).

TEM and EDS element mapping were utilized to study the detailed microstructure of the obtained products. The TEM image confirms that the material formed at 325 °C is composed of irregular small particles with pronounced graininess at the edges (Figure 3a). The HRTEM image shows that the lattice fringes with interplanar spacing of 0.2328 and 0.1746 nm correspond to the (111) plane of CuO and the (211) plane of Cu_2_O, respectively (Figure 3b). Element mapping results clearly identify the spatial distribution of Cu, O, and C in the formed CuO–Cu_2_O@C microstructures (Figure 3c–f). For the sample prepared at 450 °C, the accumulated particles are irregular (Figure 3g). The crystal plane spacing of 0.2525 nm corresponds to the (11-1) of CuO (Figure 3h). Element mapping demonstrates the existence and uniform distribution of Cu, O, and C in the obtained CuO@C microstructures (Figure 3i–l).

The growth processes of CuO–Cu_2_O@C composite and CuO@C composite can be described as follows: Firstly, Cu(Ac)_2_ begins to decompose when heated to a certain temperature, producing CH_4_, CO_2_, and CuO. The decomposition process of Cu(Ac)_2_ can be expressed as the following:

(1) $$2Cu{\left(CH_{3}COO\right)}_{2}\overset{\Delta }{\to }2CuO+3CH_{4}+3CO_{2}+2C$$

In addition, CH_4_ can be used as a reductant due to the presence of carbon and hydrogen elements. Methane acts as a reducing agent and reacts with copper oxide to generate Cu_2_O, carbon, and water under heating conditions. The oxidation–reduction process can be expressed as the following:

(2) $$4CuO+CH_{4}\overset{\Delta }{\to }2Cu_{2}O+2H_{2}O+C$$

The above process indicates that the formation of Cu_2_O is accompanied by the generation of carbon. Therefore, the carbon content in the CuO–Cu_2_O@C composite will be higher than that in the CuO@C composite.

For the sample prepared at a heating temperature of 325 °C, the NaCl column remains basically intact after baking, which means the CH_4_ formed can react with CuO during the decomposition process of Cu(Ac)_2_, so the formed sample contains Cu_2_O. As the heating temperature increases to 450 °C, the outer surface of the NaCl column presents some crevices, which means the CH_4_ formed during the decomposition process of Cu(Ac)_2_ will be released. So, the oxidation–reduction process between CH_4_ and CuO will not occur; the formed product does not contain Cu_2_O. In addition, the higher heating temperature will lead to the melting and regrowth of nanoparticles, forming irregular aggregates and porous microstructures.

The existence of carbon in formed products was identified by the Raman spectrum (Figure S1a,b). G- and D-bands are characteristic of graphitic materials [10]. The G-band at ~1350 cm^−1^ comes from the stretching vibration of the C–C bond of graphitic material. The D band at ~1580 cm^−1^ is born out of the in-plane vibration of carbon atoms with dangling bonds. D-band intensity could be utilized to measure the existence of such defects in graphite structures [20]. The crystallinity of the product can be estimated by the intensity ratio of the D peak to the G peak. The crystallinity of CuO–Cu_2_O@C and CuO@C structures is 0.82 and 0.84, respectively, which shows the carbon in the formed samples has low degrees of graphitization [21]. In addition, the peaks at 293, 347, and 625 cm^−1^ are the characteristic peaks of CuO [10], while the peaks at 496 cm^−1^ and below 200 cm^−1^ come from Cu_2_O [22]. In addition, the molecular structures were studied by FT-IR spectra (Figure S1c,d). The broad peaks at 507 and 1080 cm^−1^ come from the deformation vibration of the Cu–O bond and the stretching vibration of the Cu–O bond, respectively [23]. The bond at 1640 cm^−1^ belongs to the O–H deformation vibration of adsorbed water [24,25]. These testing results prove the existence of CuO, Cu_2_O, and C in the sample prepared at 325 °C and CuO and C in the product prepared at 450 °C.

The existence of carbon was further confirmed by thermogravimetric analysis (Figure S2a,d). The weight loss of the product below 250 °C is 0.42%, mainly resulting from the removal of adsorbed water. As the temperature increases to 300 °C, the sample quality increases by 0.72%, which comes from the complete oxidation of Cu_2_O. As the heating temperature is heated to 800 °C, the weight loss rate is approximately 0.8% for CuO–Cu_2_O@C composite and 0.5% for CuO@C composite, which corresponds to the oxidation of carbon contained in the material. The encapsulation of carbon contributes to the improvement of discharge capacity. The testing results of thermogravimetric analysis, Raman spectrum, and XRD pattern can be confirmed by each other. 

The mesoporous characteristics and specific surface area of two formed samples were analyzed. The nitrogen adsorption/desorption isotherm shows that both CuO–Cu_2_O@C and CuO@C composites show a type III isotherm [17]. The hysteresis loops at 0.75–0.95 P/P_0_ for CuO–Cu_2_O@C composite and 0.2–0.9 P/P_0_ for CuO@C composite can be found (Figure S2b,e). BET-specific surface area is 5.80 m^2^ g^−1^ for the CuO–Cu_2_O@C composite and 5.73 m^2^ g^−1^ for the CuO@C composite. The curled sunflower-shaped CuO–Cu_2_O@C composite displays a macropore size distribution centered at 80 nm (Figure S2c), which can be attributed to the stacking of nanopyramids. However, the CuO–Cu_2_O@C composite does not have an obvious meso/macro pore distribution (Figure S2f). The surface structural feature of the curled sunflower-shaped microstructure favors the rapid transfer of electrolytes and electrons, alleviating volume changes in the electrochemical reaction process.

The chemical composition and elemental valence state of the two composites were determined by XPS. For the CuO–Cu_2_O@C composite, the peaks located at 284.8, 530.3, and 933.7 eV in the XPS survey spectrum (Figure S3a) correspond to C 1s, O 1s, and Cu 2p, respectively. The fitting peaks at 284.8, 286.2, and 288.4 eV in the C 1s spectrum (Figure S3b) correspond to C–C, C–O, and C=O bonds of composites, respectively [26]. The fitting peak at 529.8 eV in the O 1s spectrum (Figure S3c) corresponded to lattice oxygen within CuO and Cu_2_O; the other two fitting peaks at 531.3 and 533.3 eV originated from the defective oxygen and adsorbed oxygen specimens, respectively [27]. In addition, the Cu 2p spectrum can be deconvoluted into Cu 2p_1/2_ and Cu 2p_3/2_ (Figure S3d); the fitting peaks at 934.7 and 956.2 eV belong to the Cu^2+^ 2p_3/2_ and Cu^2+^ 2p_1/2_, while the peaks at 932.8 and 953.7 eV correspond to the Cu^+^ 2p_3/2_ and Cu^+^ 2p_1/2_, respectively [28,29,30]. In addition, the satellite peaks at 962.4, 944.3, and 941.9 eV verify the presence of Cu^2+^ [31]. For the CuO@C composite, XPS testing results show that the existing forms of C and O elements are consistent with the curled sunflower-shaped CuO–Cu_2_O@C composite, while only Cu^2+^ can be confirmed (Figure S3e–h). The results of XPS and XRD tests can be consistent with each other.

### 3.2. Electrochemical Performance Analysis

**Figure 4:**
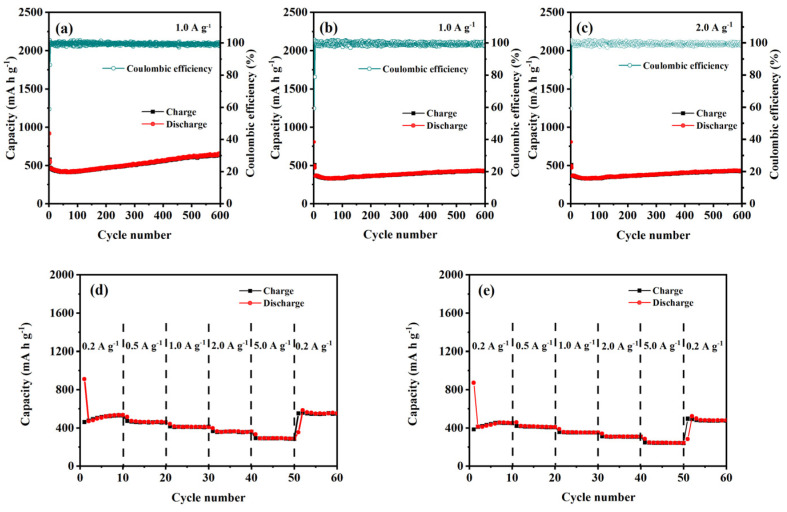
Cyclic performance and rate performance testing of CuO–Cu_2_O@C composite (**a**,**c**,**d**) and CuO@C composite (**b**,**e**).

The cyclic stability of the formed CuO–Cu_2_O@C composite and CuO@C composite was tested at a current density of 1.0 A g^−1^. For the first three cycles, a current density of 0.1 A g^−1^ was used to activate the electrode. For the CuO–Cu_2_O@C composite electrode (Figure 4a), the first charge/discharge capacity is 539.2/917.4 mA h g^−1^. In the second and third cycles, the discharge capacities change into 549.7 and 570.4 mA h g^−1^. Then, the discharge capacity decreases to 426.1 mA h g^−1^ at the 60th cycle. After that, the discharge capacities slowly increased along with the increased cycling number. After 600 cycles, the discharge capacity obtained is 647.4 mA h g^−1^. The irreversible electrochemical reactions and structural transformation during Li^+^ insertion/extraction processes can be used to explain the capacity loss during the initial cycles [11,32]. The increased discharge capacities may be caused by the further activation of electrode materials during the cycling [11]. For the CuO@C composite electrode (Figure 4b), the charge/discharge capacity in the first cycle is only 420/796.8 mA h g^−1^. In the following two cycles, the discharge capacity sharply decreased to 430.9 and 448.2 mA h g^−1^. Even with the activation effect of the electrode, its discharge capacity can only be maintained at around 450 mA h g^−1^ in the next 600 cycles of testing. Compared with single copper oxide, mixed-valent copper oxide has excellent electrochemical performance, and the existence of a carbon layer enhances this advantage.

Furthermore, the CuO–Cu_2_O@C composite electrode exhibits stable cycling performance at a high current density of 2.0 A g^−1^ (Figure 4c), and a stable discharge capacity of 431.8 mA h g^−1^ can be achieved after 600 cycles, which is also superior to the previously reported materials containing copper oxide.

The reversibility is one of the key parameters for evaluating LIBs [33]. The rate performance of CuO–Cu_2_O@C composite and CuO@C composite at various current densities (0.2, 0.5, 1.0, 2.0, and 5.0 A g^−1^) was tested between 0.01 V and 3.0 V to evaluate the stability of the formed LIBs. For the CuO–Cu_2_O@C composite electrode (Figure 4d), a discharge capacity of 530.9 mA h g^−1^ can be reached at a current density of 0.2 A g^−1^. As the current density is set at 0.5, 1.0, 2.0, and 5.0 A g^−1^, the CuO–Cu_2_O@C composite delivers a discharge capacity of 461.9, 411.6, 360.6, and 290.5 mA h g^−1^, respectively. As the current density comes back to 0.2 A g^−1^, the discharge capacity reaches 551.9 mA h g^−1^ and maintains good stability, which indicates that the CuO–Cu_2_O@C composite electrode can be gradually activated and possesses significant reversibility [34]. For the CuO@C composite electrode (Figure 4e), the discharge capacity is 455.6 mA h g^−1^ at a current density of 0.2 A g^−1^. As the current density changes from 0.5, 1.0, 2.0, and 5.0 to 0.2 A g^−1^, the discharge capacity of 411.3, 353.8, 309.9, 244.1, and 480.6 mA h g^−1^ can be obtained, respectively. The results prove that the CuO–Cu_2_O@C composite possesses better rate performance than that of the CuO@C composite.

**Figure 5:**
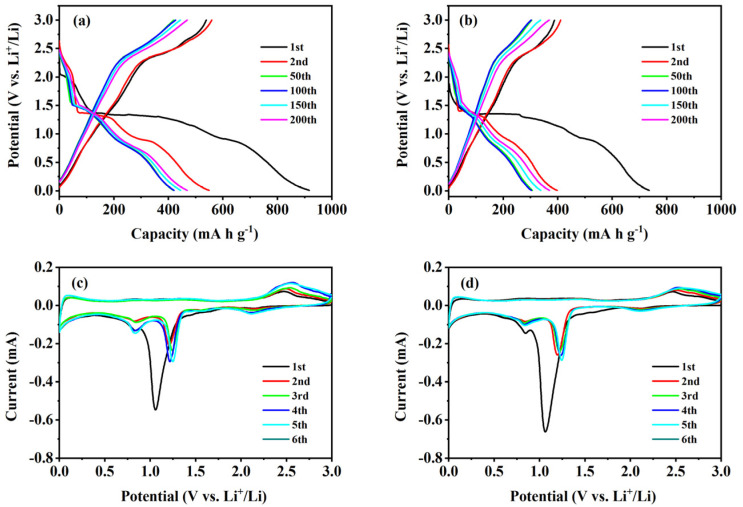
GCD curves and CV curves of CuO–Cu_2_O@C composite (**a**,**c**) and CuO@C composite (**b**,**d**).

GCD curves tested at 1.0 A g^−1^ for the 1st, 2nd, 50th, 100th, 150th, and 200th cycles were analyzed to study the electrochemical mechanism of the two composites (Figure 5a,b). For the CuO–Cu_2_O@C composite, the initial discharge curve has two small voltage platforms; the sharp peak located around 1.28 V arises from the formation of the Cu_2_O phase, while the transition of Cu_2_O to Cu occurs at 0.88 V [10]. In the subsequent discharge curve, only one long voltage platform appears at 1.41 V. In the charging curve, one voltage platform at 2.41 V can be observed. A multiphase transition between CuO and lithium can be confirmed by the constant slope with several small plateaus [35]. Since the second cycle, the voltage distribution has remained basically unchanged, showing good electrochemical stability. The GCD test results agree well with the CV test results and further indicate that mixed-valence copper-containing oxides are in favor of the insertion of lithium ions [6]. For the CuO@C composite, the platform voltages during the cycling basically coincide with those of the CuO–Cu_2_O@C composite, but the specific capacities are lower than those of the CuO–Cu_2_O@C composite. The results show that the CuO–Cu_2_O@C composite electrode has the same Li^+^ insertion/extraction mechanism with CuO@C composite, and the mixed-valence copper oxides are more conducive to the insertion of lithium ions [6].

The first six CV curves of two composites were tested at a scanning rate of 0.1 mV s^−1^. For the CuO–Cu_2_O@C composite electrode (Figure 5c), the initial three reduction peaks at 2.12, 1.05, and 0.83 V arise from the reduction process of CuO to Cu^II^_1−x_Cu^I^_x_O_1−x/2_ (0 ≤ x ≤ 0.4) solid solution mixed phase and then to Cu phase in lithium oxide matrix [30,36]. The decomposition of the electrolyte solution will lead to the appearance of inorganic by-products and solid electrolyte interface (SEI) [37]. Previous studies have reported the appearance of a cuprous oxide mesophase [38]. In the following cycle, the reduction peaks appear at 2.10, 1.21, and 0.82 V. The decreased peak intensity and integral area correspond to the irreversible capacity loss [39,40]. The oxidation peak that appears at 2.45 V comes from the reaction of Cu oxidation to Cu_2_O and further oxidation to CuO [39]. Due to the small structural changes in copper-based oxides, the oxidation peak position at 2.45 V slightly increased to 2.49 V in the following cycles. In the subsequent cycle, the CV curves are basically overlapped, which indicates that the curled sunflower-shaped CuO–Cu_2_O@C composite provides excellent reversibility in electrochemical reactions [22]. For the CuO@C composite electrode (Figure 5d), the reduction peak current value around 1.05 V is larger than that of the CuO–Cu_2_O@C composite electrode, which is mainly born out of the reduction transformation of more Cu^2+^ to Cu^+^. The chemical reactions occurring in the oxidation–reduction process are as follows [22,27]:

(3) $$2CuO+2xLi^{+}+2xe^{-}\to 2Cu_{1-x}^{II}Cu_{x}^{I}O_{1-x/2}+xLi_{2}O$$

(4) $$2Cu_{1-x}^{II}Cu_{x}^{I}O_{1-x/2}+2\left(1-x\right)Li^{+}+2\left(1-x\right)e^{-}\to Cu_{2}O+\left(1-x\right)Li_{2}O$$

(5) $$Cu_{2}O+2Li^{+}+2e^{-}\to 2Cu+Li_{2}O$$

(6) $$Cu+Li_{2}O\to Cu+2Li^{+}+2e^{-}$$

In this work, EIS before cycles were analyzed. For the Nyquist plot and the fitting circuit of CuO–Cu_2_O@C composite and CuO@C composite (Figure S4), the intercept on the Z-real axis corresponds to the electrolyte solution resistance (R_s_); the semicircle in the high and medium frequency range can be attributed to the charge transfer resistance (R_ct_); and the slope in the low-frequency region reflects the independent Li^+^ diffusion behavior [41]. A constant-phase element (CPE) is used in the equivalent circuit due to the inhomogeneity of the working electrode surface [42]. The fitting results have been presented and analyzed (Table S1). For the CuO–Cu_2_O@C composite electrode, the R_s_ and R_ct_ values are 3.4 Ω and 128.5 Ω, respectively. For the CuO@C composite electrode, the R_s_ and R_ct_ values are 2.9 Ω and 200.3 Ω, respectively. Furthermore, the slope of Nyquist plots in the low-frequency region shows that the diffusion resistance of CuO–Cu_2_O@C composite is lower than that of CuO@C composite. So, the higher charge/discharge capacity of the CuO–Cu_2_O@C composite electrode than that of the CuO@C composite electrode is related to better charge transfer and Li^+^ diffusion properties. The existence of cuprous oxide in the composite accelerates the electrode oxidation reaction, resulting in an improvement in charge transfer performance. The presence of carbon forms a permeable conductive pathway that facilitates charge separation, accelerates ion transfer, and facilitates diffusion of electrolytes during the reaction process, enhancing the electrochemical conductivity and the structural robustness of electrode material, resulting in excellent lithium storage performance.

**Figure 6:**
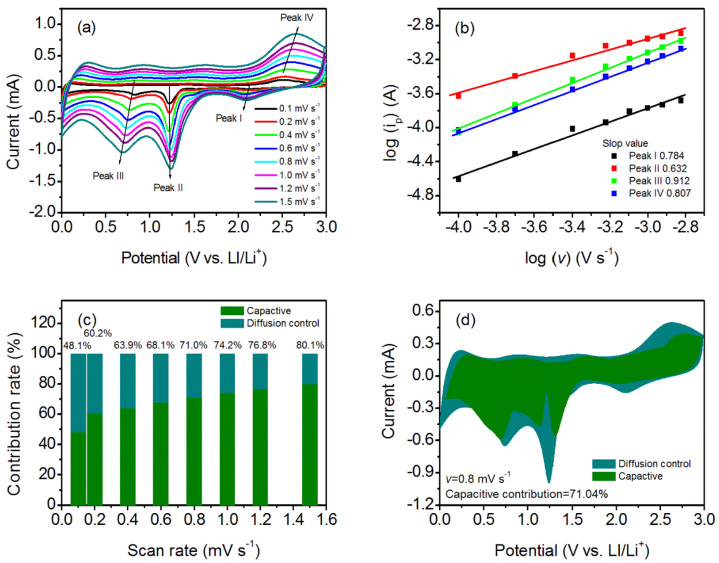
(**a**) CV curves of the fresh half cells at different scan rates; (**b**) determination of the *b* value by the power law relationship; (**c**) quantitation of capacity contribution at different scan rates; and (**d**) CV curves for the curled sunflower-shaped CuO–Cu_2_O@C composite electrode at a scan rate of 0.8 mV s^−1^. The shaded region is the capacitive current contribution.

Furthermore, the CV curves of the fresh half cells with different scanning rates were recorded to study the electrochemical kinetics of the curled sunflower-shaped CuO–Cu_2_O@C composite (Figure 6a). The power law relationship between current (*i*) and scanning rate (*v*) was utilized to evaluate the capacitance effect [43,44,45]:

(7) $$i=av^{b}$$

In the above equation, *b* reflects the determining electrochemical process. The *b* value near 1.0 means the electrochemical process is pseudocapacitive-controlled [43,46,47]. Normally, the *b* value is calculated by the slope of log(*i*) versus log(*v*) for the different redox peaks. For the curled sunflower-shaped CuO–Cu_2_O@C composite electrode, the obtained *b* values of 0.784, 0.632, 0.912, and 0.807 for peaks I–IV mean that the electrochemical process is surface-controlled (Figure 6b). The surface-controlled process will contribute to fast kinetics, ensuring excellent reversible cycling properties and rate performance [48,49]. Moreover, the current response at a certain potential may be distinguished into a surface-capacitive effect (*k*_1_*v*) and a diffusion-controlled process (*k_2_ v*^1/2^) according to the following equation:

(8) $$i\left(V\right)=k_{1}v+k_{2}v^{1/2}$$

The results suggest that the pseudocapacitive contribution percentage increases along with the increased scanning rate (Figure 6c). As the scanning rate increases to 1.5 mV s^−1^, the contribution of the pseudocapacitance effect is 80.1%. CV curve at a scanning rate of 0.8 mV s^−1^ is analyzed; the pseudocapacitive contribution is 71.0% (Figure 6d). Therefore, the excellent electrochemical properties of the formed CuO–Cu_2_O@C composite at high testing current density is related to the contribution of pseudocapacitive behavior, which favors a fast Faradaic surface charge transfer reaction.

## 4. Conclusions

In brief, a bilayer salt bath baking method was designed for the preparation of a mixed-valence copper oxide composite. As the anode material of LIBs, the formed CuO–Cu_2_O@C composite exhibits excellent cycle stability (431.8 mA h g^−1^ at 2.0 A g^−1^ over 600 cycles). The excellent cycle performance can be attributable to the formation of a mixed-valence copper oxide@C composite and the pseudocapacitive effect. The existence of cuprous oxide improves the conductivity of electrode material; the presence of carbon forms a permeable conductive pathway that facilitates charge separation, ion transfer, and electrolyte diffusion, enhancing the electrochemical conductivity and the structural robustness of electrode material, resulting in excellent lithium storage performance.

## Data Availability

Data is contained within the article and Supplementary Material.

## Supplementary Materials

The following supporting information can be downloaded at: https://www.mdpi.com/article/10.3390/nano14171378/s1.

## References

[1] Chen J., Bai Z., Li X., Wang Q., Du J., Lu R., Liu X. (2022). In-situ synthesis of reduced graphene oxide wrapped Mn_3_O_4_ nanocomposite as anode materials for high-performance lithium-ion batteries. Ceram. Int..

[2] Choi C., Ashby D.S., Butts D.M., DeBlock R.H., Wei Q., Lau J., Dunn B. (2019). Achieving high energy density and high power density with pseudocapacitive materials. Nat. Rev. Mater..

[3] Gou W., Kong X., Wang Y., Ai Y., Liang S., Pan A., Cao G. (2019). Yolk-shell structured V_2_O_3_ microspheres wrapped in N, S co-doped carbon as pea-pod nanofibers for high-capacity lithium ion batteries. Chem. Eng. J..

[4] Trukawka M., Wenelska K., Singer L., Klingeler R., Chen X., Mijowska E. (2021). Hollow carbon spheres loaded with uniform dispersion of copper oxide nanoparticles for anode in lithium- ion batteries. J. Alloys Compd..

[5] Yan B.-L., Jun D., Wang J., Yang T., Mao X.-H. (2022). A simplified electrophoretic deposition route for sandwiched structure-based Mn_3_O_4_/G composite electrodes as high-capacity anodes for lithium-ion batteries. J. Alloys Compd..

[6] Dong X., Dong F., Zhang Y., Fu C., Cui C., Wang L., Zeng S. (2022). Preparation of V_2_O_5_ porous microstructures with enhanced performances of lithium ion batteries. Mater. Chem. Phys..

[7] Lee Y.-T., Kuo C.-T., Yew T.-R. (2021). Investigation on the Voltage Hysteresis of Mn_3_O_4_ for Lithium-Ion Battery Applications. ACS Appl. Mater. Interfaces.

[8] Yan P., Ji L., Liu X., Guan Q., Guo J., Shen Y., Zhang H., Wei W., Cui X., Xu Q. (2021). 2D amorphous-MoO_3_−x@Ti_3_C_2_-MXene non-van der Waals heterostructures as anode materials for lithium-ion batteries. Nano Energy.

[9] Zhang C., Chen Z., Wang H., Nie Y., Yan J. (2021). Porous Fe_2_O_3_ Nanoparticles as Lithium-Ion Battery Anode Materials. ACS Appl. Nano Mater..

[10] Zhang Y., Liu W., Zhu Y., Zhang Y., Zhang R., Li K., Liu G. (2021). Facile Self-Assembly Solvothermal Preparation of CuO/Cu_2_O/Coal-Based Reduced Graphene Oxide Nanosheet Composites as an Anode for High-Performance Lithium-Ion Batteries. Energy Fuels.

[11] Xu Y., Chu K., Li Z., Xu S., Yao G., Niu P., Zheng F. (2020). Porous CuO@C composite as high-performance anode materials for lithium-ion batteries. Dalton Trans..

[12] Dong Y., Jiang X., Mo J., Zhou Y., Zhou J. (2020). Hollow CuO nanoparticles in carbon microspheres prepared from cellulose-cuprammonium solution as anode materials for Li-ion batteries. Chem. Eng. J..

[13] Kong Y., Jiao R., Li H., Xu S., Cui C., Zeng S., Wang L. (2020). Enhanced lithium storage performance of binary cooperative complementary CuO-Mn_3_O_4_ nanocomposites directly synthesized by hydrothermally controlled method. J. Alloys Compd..

[14] Hossain S., Abdalla A.M., Suhaili S.B., Kamal I., Shaikh S.P., Dawood M.K., Azad A.K. (2020). Nanostructured graphene materials utilization in fuel cells and batteries: A review. J. Energy Storage.

[15] Song X., Zhu J., Hu G. (2021). Facile one-step synthesis of three-dimensional porous Cu_2_O electrode for lithium-ion batteries. Mater. Lett..

[16] Sun Y., Zhang P., Wang B., Wu J., Ning S., Xie A., Shen Y. (2018). Hollow porous CuO/C nanorods as a high-performance anode for lithium ion batteries. J. Alloys Compd..

[17] Lin X., Lin J., Niu J., Lan J., Reddy R.C.K., Cai Y., Liu J., Zhang G. (2018). *In situ* synthesis of Cu_2_O–CuO–C supported on copper foam as a superior binder-free anode for long-cycle lithium-ion batteries. Mater. Chem. Front..

[18] Jiao R., Xiao X., Zhou S., Zhu K., Zhang Y., Wei D., Zeng S. (2022). Solid-State Fabrication of Co_3_V_2_O_8_@C Anode Materials with Outstanding Rate Performance and Cycling Stability by Synergistic Effects of Pseudocapacity and Carbon Coating. J. Phys. Chem. C.

[19] Hu L., Huang Y., Zhang F., Chen Q. (2013). CuO/Cu_2_O composite hollow polyhedrons fabricated from metal–organic framework templates for lithium-ion battery anodes with a long cycling life. Nanoscale.

[20] Pol V.G., Thackeray M.M. (2011). Spherical carbon particles and carbon nanotubes prepared by autogenic reactions: Evaluation as anodes in lithium electrochemical cells. Energy Environ. Sci..

[21] Zhu S., Li J., He C., Zhao N., Liu E., Shi C., Zhang M. (2015). Soluble salt self-assembly-assisted synthesis of three-dimensional hierarchical porous carbon networks for supercapacitors. J. Mater. Chem. A.

[22] Hao Z., Qin M., Li Y., Lv X., Zhang D., Wang Q. (2019). Carbon Nano-Onions Embedded CuO Nanosheets: An Excellent Stable Anode Material for Lithium Ion Battery. IOP Conf. Ser. Mater. Sci. Eng..

[23] Song Y.Z., Liu Z.J., Qi B.X., Li M., Xie J., Song W.H. (2021). Facile Synthesis of Micro CuO Crystals for Li Ion Full Battery. J. Inorg. Organomet. Polym. Mater..

[24] Dong F., Dong X., Xu S., Li H., Zeng S., Fu C., Wang L. (2021). Study for the preparation of Cu^2+^-doped twin spherical MnCO_3_ structure as an anode material for high-performance lithium-ion batteries. CrystEngComm.

[25] Sekhar S.C., Nagaraju G., Yu J.S. (2018). Ant-cave structured MnCO_3_/Mn_3_O_4_ microcubes by biopolymer-assisted facile synthesis for high-performance pseudocapacitors. Appl. Surf. Sci..

[26] Zhang R., Li X., Ni L., Xie A., Li P., Shen Y., Lao L. (2020). Octagonal Flower-like CuO/C/NF Nanocomposite as a Self-Supporting Anode for High-Performance Lithium-Ion Batteries. ChemElectroChem.

[27] Liu S., Hou H., Liu X., Duan J., Yao Y., Liao Q. (2016). High-performance hierarchical cypress-like CuO/Cu_2_O/Cu anode for lithium ion battery. Ionics.

[28] Hu P., Meng C., Li F., Wang P., Zhou H., Li X., Yuan A. (2022). Hierarchical multi-yolk-shell copper oxide@copper-1, 3, 5-benzenetricarboxylate as an ultrastable anode for lithium ion batteries. J. Colloid Interface Sci..

[29] Ma T., Gao L., Liu Y., Zhang L., Yang X. (2021). Porous CuO/Cu_2_O heterostructured arrays as anode for high-performance sodium-ion batteries. Ionics.

[30] Wang Y., Cao L., Li J., Kou L., Huang J., Feng Y., Chen S. (2020). Cu/Cu_2_O@Ppy nanowires as a long-life and high-capacity anode for lithium ion battery. Chem. Eng. J..

[31] Yuan W., Ye Y., Yang Y., Zhang X., Pan B., Peng Z., Wu M., Qiu Z., Wang C., Yuan Y. (2020). CuO nanoflowers/copper fiber felt integrated porous electrode for lithium-ion batteries. Sci. China Technol. Sci..

[32] Gao G., Lu S., Dong B., Xiang Y., Xi K., Ding S. (2016). Mesoporous Co_3_V_2_O_8_nanoparticles grown on reduced graphene oxide as a high-rate and long-life anode material for lithium-ion batteries. J. Mater. Chem. A.

[33] Li X., Wu G., Liu X., Li W., Li M. (2017). Orderly integration of porous TiO_2_(B) nanosheets into bunchy hierarchical structure for high-rate and ultralong-lifespan lithium-ion batteries. Nano Energy.

[34] Yu L.-Q., Zhao S.-X., Wu X., Wu Q.-L., Li J.-W., Zhao E.-L. (2019). Effects of vanadium pentoxide with different crystallinities on lithium ion storage performance. CrystEngComm.

[35] Liu Y., Wang W., Gu L., Wang Y., Ying Y., Mao Y., Sun L., Peng X. (2013). Flexible CuO Nanosheets/Reduced-Graphene Oxide Composite Paper: Binder-Free Anode for High-Performance Lithium-Ion Batteries. ACS Appl. Mater. Interfaces.

[36] Wang L.-H., Gao S., Ren L.-L., Zhou E.-L., Qin Y.-F. (2021). The Synergetic Effect Induced High Electrochemical Performance of CuO/Cu2O/Cu Nanocomposites as Lithium-Ion Battery Anodes. Front. Chem..

[37] Banerjee A., Singh U., Aravindan V., Srinivasan M., Ogale S. (2013). Synthesis of CuO nanostructures from Cu-based metal organic framework (MOF-199) for application as anode for Li-ion batteries. Nano Energy.

[38] Sahay R., Kumar P.S., Aravindan V., Sundaramurthy J., Ling W.C., Mhaisalkar S.G., Ramakrishna S., Madhavi S. (2012). High Aspect Ratio Electrospun CuO Nanofibers as Anode Material for Lithium-Ion Batteries with Superior Cycleability. J. Phys. Chem. C.

[39] Li Z., Xie G., Wang C., Liu Z., Chen J., Zhong S. (2021). Binder free Cu_2_O/CuO/Cu/Carbon-polymer composite fibers derived from metal/organic hybrid materials through electrodeposition method as high performance anode materials for lithium-ion batteries. J. Alloys Compd..

[40] Xu C., Manukyan K.V., Adams R.A., Pol V.G., Chen P., Varma A. (2019). One-step solution combustion synthesis of CuO/Cu_2_O/C anode for long cycle life Li-ion batteries. Carbon.

[41] Rai A.K., Anh L.T., Gim J., Mathew V., Kang J., Paul B.J., Singh N.K., Song J., Kim J. (2013). Facile approach to synthesize CuO/reduced graphene oxide nanocomposite as anode materials for lithium-ion battery. J. Power Sources.

[42] Yan B., Li X., Bai Z., Zhao Y., Dong L., Song X., Li D., Langford C., Sun X. (2016). Crumpled reduced graphene oxide conformally encapsulated hollow V_2_O_5_ nano/microsphere achieving brilliant lithium storage performance. Nano Energy.

[43] Li Y., Zhang R., Zhou W., Wu X., Zhang H., Zhang J. (2019). Hierarchical MoS_2_ Hollow Architectures with Abundant Mo Vacancies for Efficient Sodium Storage. ACS Nano.

[44] Liu G., Huang M., Zhang Z., Xi B., Li H., Xiong S. (2021). Robust S-doped TiO2@N,S-codoped carbon nanotube arrays as free-binder anodes for efficient sodium storage. J. Energy Chem..

[45] Wang Y., Wu H., Liu Z., Zhao H., Huang L., Wang Q., Liu H., Zhang Y. (2019). Tailoring sandwich-like CNT@MnO@N-doped carbon hetero-nanotubes as advanced anodes for boosting lithium storage. Electrochim. Acta.

[46] Hareendrakrishnakumar H., Chulliyote R., Joseph M.G. (2018). Micro- and Nanocrystalline Inverse Spinel LiCoVO_4_ for Intercalation Pseudocapacitive Li^+^ Storage with Ultrahigh Energy Density and Long-Term Cycling. ACS Appl. Energy Mater..

[47] Zhang Y., Ding Z., Foster C.W., Banks C.E., Qiu X., Ji X. (2017). Oxygen Vacancies Evoked Blue TiO_2_(B) Nanobelts with Efficiency Enhancement in Sodium Storage Behaviors. Adv. Funct. Mater..

[48] Chen Z., Li S., Zhao Y., Aboud M.F.A., Shakir I., Xu Y. (2019). Ultrafine FeS_2_ nanocrystals/porous nitrogen-doped carbon hybrid nanospheres encapsulated in three-dimensional graphene for simultaneous efficient lithium and sodium ion storage. J. Mater. Chem. A.

[49] Yin M., Feng X., Zhao D., Zhao Y., Li H., Zhou W., Liu H., Bai X., Wang H., Feng C. (2019). Hierarchical Co_9_S_8_@Carbon Hollow Microspheres as an Anode for Sodium Ion Batteries with Ultralong Cycling Stability. ACS Sustain. Chem. Eng..

