# Supplementary material for: Solid-State Construction of CuO–Cu2O@C with Synergistic Effects of Pseudocapacity and Carbon Coating for Enhanced Electrochemical Lithium Storage
Source: Nanomaterials (Basel). 2024 Aug 23;14(17):1378. doi: 10.3390/nano14171378 (PMC11397226; doi:10.3390/nano14171378)
Supplement: Supplementary file 1 [file nanomaterials-14-01378-s001.zip › nanomaterials-3124248-supplementary.pdf]

Supporting Information

Solid-State Construction of CuO–Cu_2O@C with Synergistic Effects of Pseudocapacity and Carbon Coating for Enhanced Electrochemical Lithium Storage

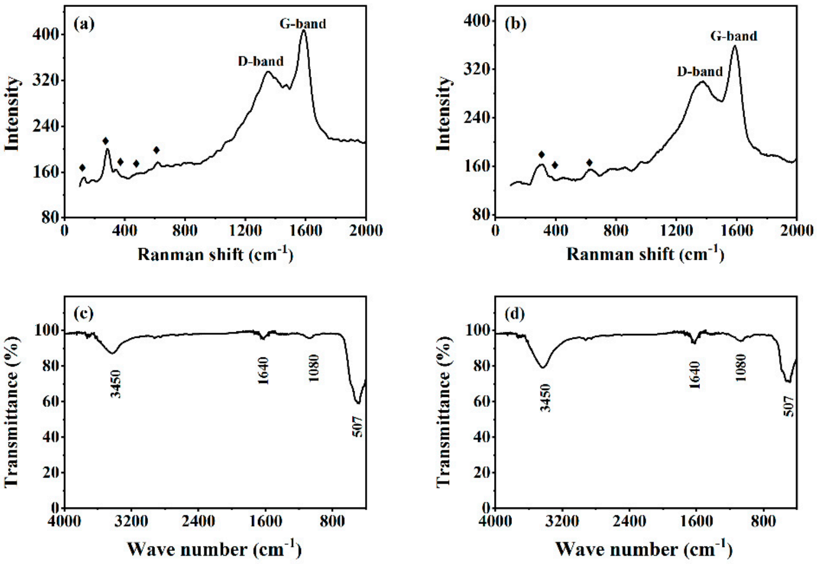

Figure S1 Raman spectra and FT-IR spectra of CuO-Cu_2O@C composite (a,c) and CuO@C composite (b,d).

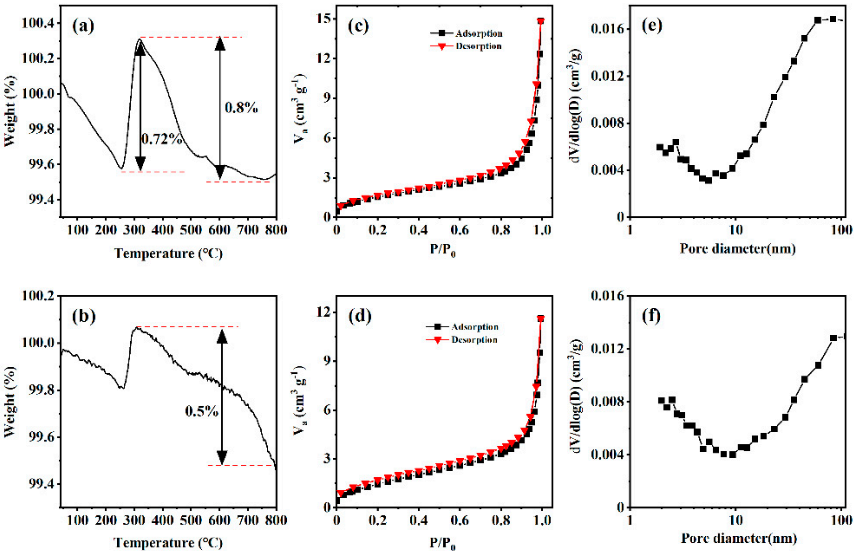

Figure S2 TGA curves, Isothermal adsorption-desorption curves and corresponding BJH pore size distributions of CuO-Cu_2O@C composite (a,c,e) and CuO@C composite (b,d,f).

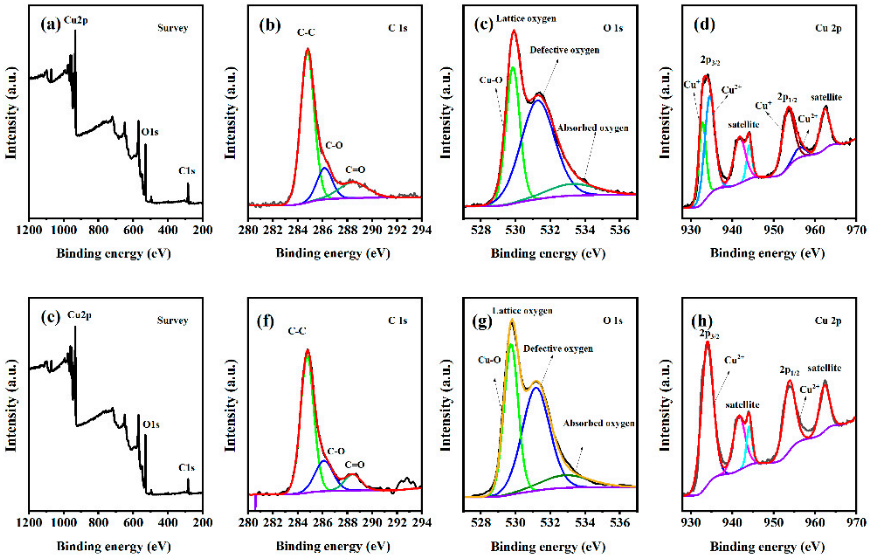

Figure S3 XPS spectra of CuO-Cu_2O@C composite (a-d) and CuO@C composite (e-h).

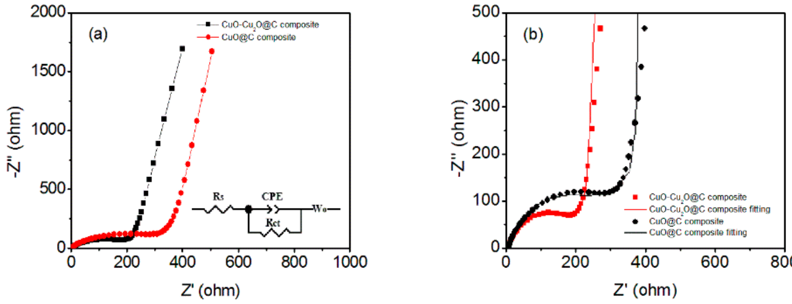

Figure S4 Nyquist plots of CuO-Cu_2O@C composite and CuO@C composite before cycling (a) and the enlarged fitting plots (b). The inset shows the corresponding equivalent electrical circuit.

Table S1: Numerical values associated with the equivalent circuit elements and the error between the raw and fitted

Sample	Element	Value	Error	Error%
CuO-Cu_2O@C composite	R_s	3.398	0.19041	5.6036
	CPE1-T	1.1572E-5	2.2312E-06	19.281
	CPE1-P	0.89287	0.023821	2.6679
	R_{ct}	128.5	8.8524	6.889
	Z_w-R	278.3	41.95	15.074
	Z_w-T	0.075051	0.012773	17.019
	Z_w-P	0.48085	0.0082268	1.7111
CuO@C composite	R_s	2.883	0.23893	8.2875
	CPE1-T	1.4985E-05	2.8503E-06	19.021
	CPE1-P	0.85666	0.024477	2.8573
	R_{ct}	200.3	14.536	7.2571
	Z_w-R	525.1	71.976	13.707
	Z_w-T	0.15397	0.023202	15.069
	Z_w-P	0.50056	0.011446	2.2866
